# Assessment of carbapenemase genes and antibiotic resistance profiles in ceftazidime–avibactam resistant *Klebsiella pneumoniae* isolates: A single-center cross-sectional study

**DOI:** 10.1097/MD.0000000000045289

**Published:** 2025-10-10

**Authors:** Ipek Koçer, Hadiye Demirbakan

**Affiliations:** a Department of Medical Microbiology, Faculty of Medicine, Sanko University, Gaziantep, Turkey.

**Keywords:** antibiotic resistance, carbapenem resistance, *Klebsiella pneumoniae*, NDM gene, OXA-48 gene, Turkey

## Abstract

**Background:**

Carbapenem-resistant *Klebsiella pneumoniae* (CRKp) is an urgent global health threat due to its rapid spread and limited treatment options. Ceftazidime–avibactam exhibits broad efficacy against gram-negative bacteria, including CRKp; however, emerging resistance to this agent is increasingly reported. Understanding the prevalence of ceftazidime–avibactam resistance and the underlying carbapenemase genes is critical for optimizing antimicrobial stewardship and guiding clinical management. This study aimed to determine the prevalence of ceftazidime avibactam resistance among CRKp isolates collected from various clinical specimens, and to analyze their associated carbapenemase genes and antibiotic resistance profiles.

**Methods:**

This cross-sectional study analyzed 312 *K pneumoniae* isolates obtained from various clinical specimens of hospitalized patients at a tertiary care hospital in Turkey. Antibiotic susceptibility testing was performed using the disk diffusion method for ceftazidime–avibactam and broth microdilution for both colistin and ceftazidime–avibactam. Molecular detection of carbapenemase genes was carried out using polymerase chain reaction.

**Results:**

Ceftazidime–avibactam resistance was identified in 21.5% (67/312) of CRKp isolates. Among these isolates, 37.3% harbored both OXA-48 and NDM genes, 13.4% carried NDM alone, 10.4% carried OXA-48 alone, and 38.8% lacked these genes. The majority of resistant isolates originated from urine (31.3%), followed by tracheal aspirate (29.9%), and blood (22.4%) specimens. The prevalence of colistin susceptibility among ceftazidime–avibactam-resistant CRKp isolates was 56.7%.

**Conclusions:**

The coexistence of NDM and OXA-48 genes is a major contributor to ceftazidime–avibactam resistance in CRKp isolates, particularly in urinary and respiratory tract infections. These findings underscore the need for ongoing surveillance and tailored antibiotic stewardship programs to control the spread of resistance in hospital settings.

## 1. Introduction

The growing complexity of infections necessitates the development of innovative and effective solutions.^[1,2]^ The slow pace of antimicrobial development coupled with the rapid emergence and spread of resistant organisms highlights the critical importance of optimizing the use of existing antimicrobials through antimicrobial stewardship (AMS). The widespread use of carbapenem antibiotics in recent years has led to increasing resistance among gram-negative bacteria, underscoring the urgent need for new antimicrobial agents.^[3]^

Carbapenemase-producing enterobacterales represent a significant global health threat due to their frequent multidrug-resistant (MDR) phenotypes, which critically limit treatment options.^[4]^ Carbapenemases are classified into 3 Ambler classes (A, B, and D), with the most clinically relevant enzymes being *Klebsiella pneumoniae* carbapenemase (KPC; class A), New Delhi metallo-β-lactamase (NDM; class B), and oxacillinase-48 (OXA-48)-like enzymes (class D).^[5]^ NDM producers have spread rapidly worldwide, largely due to international travel and trade.^[6]^

Since the first description of the OXA-48 carbapenemase in Turkey in 2008, OXA-48 producers have been extensively reported in nosocomial and community outbreaks in many parts of the word, particularly in the Mediterranean area and European countries.^[7,8]^ The dissemination of these enzymes is largely driven by plasmid-mediated transmission, enabling their rapid spread among bacterial populations. Surveillance and early detection are essential to curb the spread of such resistance mechanisms, particularly in high-risk healthcare settings.^[4]^

Ceftazidime–avibactam (CAZ–AVI), a combination of the third-generation broad-spectrum cephalosporin ceftazidime and the β-lactamase inhibitor avibactam, demonstrates potent activity against gram-negative bacteria, including carbapenem-resistant *Klebsiella pneumoniae* (CRKp) and *Pseudomonas aeruginosa*, while exhibiting minimal activity against anaerobic and gram-positive bacteria.^[9,10]^ The Surgical Infection Society guidelines recommend CAZ–AVI as an alternative for empiric therapy in patients at risk of infections caused by extended-spectrum β-lactamase (ESBL)-producing and AmpC β-lactamase–producing Enterobacteriaceae.^[11]^ Likewise, the 2017 World Society of Emergency Surgery guidelines endorse CAZ–AVI use in patients with suspected or confirmed infections caused by carbapenemase-producing *K pneumoniae*.^[12]^

Given the limited therapeutic options for infections caused by carbapenemase-producing Enterobacterales, CAZ–AVI has emerged as one of the last-resort treatment options. However, despite its recent introduction, resistance to this combination has already been reported in various settings.

This study aimed to assess the resistance profiles and prevalence of carbapenemase genes in CAZ–AVI -resistant *K pneumoniae* isolates obtained from hospitalized patients in Turkey.

## 2. Materials and methods

The study was conducted in accordance with the ethical principles stated in the Declaration of Helsinki, and approval for the study was granted by the Sanko University Clinical Research Ethics Committee on June 12, 2024 (No. 2024/06). Since the study used bacterial isolates collected during routine care and did not involve direct patient intervention, informed consent was waived in accordance with ethical standards. All procedures involving bacterial isolates were performed in compliance with institutional regulations. The study followed the STROBE Checklist to ensure clear, complete, and transparent reporting of observational data.

### 2.1. Study design and setting

**Figure 1.**
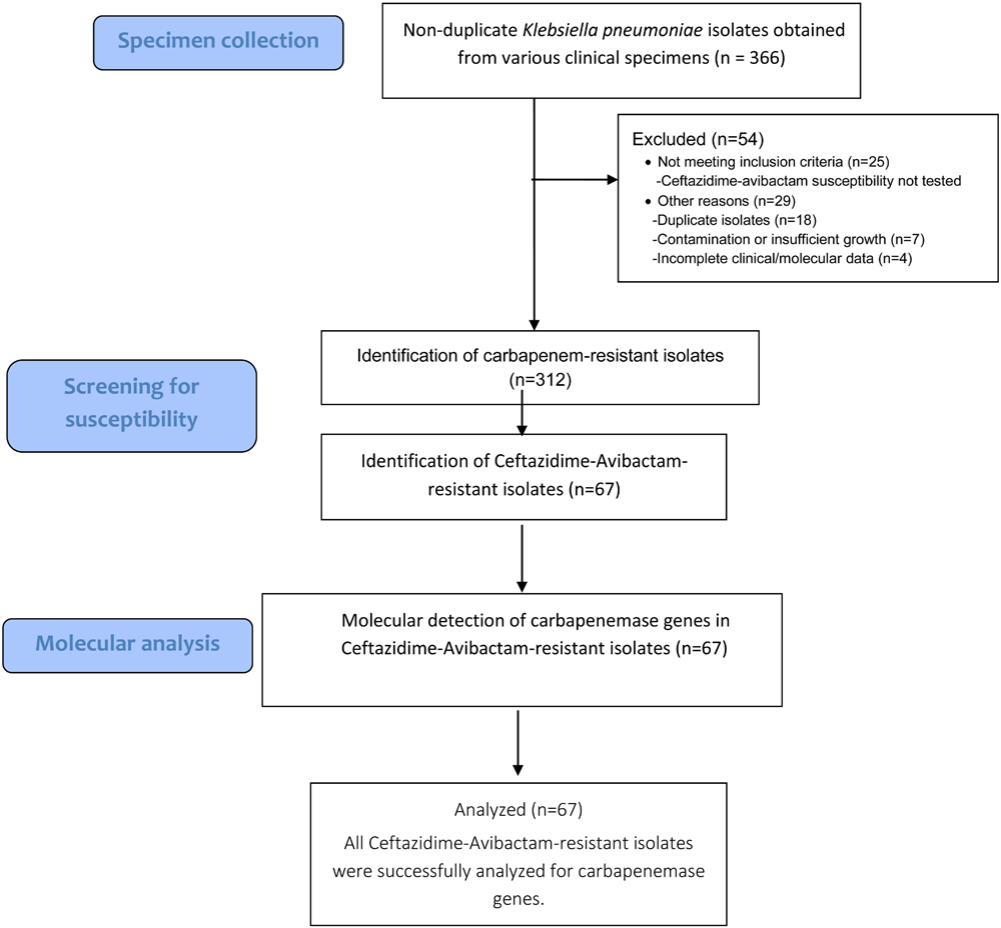
Study design and workflow.

This laboratory-based cross-sectional study was conducted at Sanko University Hospital in Gaziantep, Turkey. The study assessed the prevalence, antimicrobial resistance profiles, and molecular characteristics of CRKp isolates obtained from various clinical specimens submitted to the microbiology laboratory between November 2021 and February 2024. The study flow diagram is presented in Figure 1.

### 2.2. Participants (isolate selection and eligibility criteria)

All bacterial isolates included in the study were obtained from hospitalized patients with suspected bacterial infections during the study period. A total of 366 non-duplicate *K pneumoniae* clinical isolates were initially screened. Isolates suspected to be contaminants (e.g., mixed bacterial flora inconsistent with clinical source), repeated isolates from the same patient, or those showing insufficient growth or suggesting colonization rather than true infection were excluded. Following these criteria, 54 isolates were removed from the study. The remaining 312 isolates were identified as CRKp based on susceptibility testing and included in the final analysis. Isolates found to be resistant to CAZ–AVI (n = 67) underwent further molecular testing to detect carbapenemase genes and were also evaluated for colistin susceptibility.

Each isolate represented a distinct patient. The antibiotic treatment status of patients at the time of sample collection was not available.

### 2.3. Specimen collection and laboratory procedures

Clinical specimens included blood, urine, bronchoalveolar lavage fluid, wound, peritoneal fluid, cerebrospinal fluid, and tracheal aspirate obtained from patients with suspected bacterial infections. These specimens were inoculated on 5% sheep blood agar, chocolate agar, and MacConkey agar media (all from Becton Dickinson) and incubated at 37°C for 18 to 24 hours. Bacterial identification and susceptibility testing were performed using conventional methods and the BD Phoenix100 automated system (Becton Dickinson, BD Diagnostic Systems, Sparks). All laboratory analyses were performed by experienced microbiologists following standardized protocols to ensure data quality and minimize misclassification bias. The automated system was used to ensure standardized processing and minimize the risk of contamination.

### 2.4. Antimicrobial susceptibility testing

The presence of carbapenem resistance and susceptibility to CAZ–AVI in the CRKp isolates was determined using the Kirby–Bauer disk diffusion method. Following incubation at 37˚C for 18 to 24 hours, isolates showing inhibition zone diameters of <13 mm for CAZ–AVI were interpreted as resistant according to the EUCAST guidelines.^[13]^ Colistin susceptibility in CAZ–AVI-resistant CRKp isolates was assessed using the broth microdilution method (MIC COL, Diagnostics Inc., Slovakia). Isolates exhibiting minimum inhibitory concentration (MIC) values >2 μg/mL were classified as resistant according to EUCAST standards.^[13]^

### 2.5. Detection of carbapenem resistance genes

The presence of genes associated with carbapenem resistance, including blaKPC, blaOXA-48, blaNDM, and blaVIM/blaIMP, was investigated using conventional polymerase chain reaction methods.^[14]^

## 3. Sample size

No formal sample size calculation was performed due to the descriptive nature of the study. All eligible isolates collected during the study period were included in the analysis.

### 3.1. Statistical analysis

All collected data were double-checked for accuracy and completeness prior to the analysis. Data coding and processing were performed using the R statistical software (version 4.1.2, R Foundation for Statistical Computing, Vienna, Austria). Statistical analyses were conducted using IBM SPSS Statistics for Windows, Version 23.0 (IBM Corp., Armonk) . Mann–Whitney *U* test was used to examine the association between age and CAZ–AVI resistance. Pearson’s chi-square test was employed to evaluate associations between categorical variables. Continuous variables were assessed for normality using the Shapiro–Wilk test. Descriptive statistics were used to summarize the demographic and clinical characteristics of the cohort. Categorical variables were presented as frequencies and percentages, while continuous variables (e.g., age, colistin MIC, inhibition zones) were summarized using mean ± standard deviation or median with interquartile range (IQR), depending on their distribution. A *P*-value of <.05 was considered statistically significant.

## 4. Results

### 4.1. Demographic and clinical characteristics of the cohort

The mean age of the patients with CAZ–AVI resistance (n = 67) was 58.4 years (±20.4), and 32 (47.8%) were female. No significant association was found between age and CAZ–AVI resistance (*P* > .05). However, a significant association was observed between sex and prior carbapenem use among CAZ–AVI resistant patients (*P* < .001).

Fifteen patients (22.4%) had a history of *K pneumoniae* infection within the past 6 months. Prior exposure to CAZ–AVI was rare, documented in only 2 patients (3.0%), whereas prior carbapenem use was common, with 43 patients (64.2%) having a history of carbapenem treatment. Specifically, 24 of 35 males (68.6%) had a history of prior carbapenem exposure, compared to 19 of 32 females (59.4%).

Significant associations were observed between prior use of CAZ–AVI and diagnosis of a *K pneumoniae* infection within the past 6 months (*P* = .047), as well as between prior carbapenem use and *K pneumoniae* diagnosis in patients with CAZ–AVI resistance (*P* < .001).

The majority of patients were admitted to the Surgical Intensive Care Unit (62.7%), followed by the Department of Internal Medicine (25.4%) and the Surgical Ward (11.9%).

Invasive procedures were common among the patients: 27 (40.3%) received total parenteral nutrition, 21 (31.3%) underwent invasive mechanical ventilation, 4 (6.0%) had hemodialysis, 3 (4.5%) had tracheostomy, 3 (4.5%) had a central catheter, and 1 patient each (1.5%) had a port catheter or vacuum-assisted closure.

Regarding comorbidities, diabetes mellitus was the most frequent (37.3%), followed by hypertension (32.8%), malignancy/hemopathy (23.9%), kidney failure (19.4%), cardiovascular disease (16.4%), cerebrovascular accident (7.5%), trauma (6.0%), and demyelinating disease (4.5%).

Molecular testing of carbapenemase genes revealed that 9 isolates (13.4%) harbored the blaNDM gene, 7 isolates (10.4%) carried the blaOXA-48 gene, and 25 isolates (37.3%) exhibited coexistence of both blaNDM and blaOXA-48 genes. Notably, 26 isolates (38.8%) were negative for both genes (Table 1).

**Table 1:** Demographic and clinical characteristics of the cohort (n = 67).

Variable	n (%) or mean ± SD
Age (yr)	58.4 **±** 20.4
Sex, female	32 (47.8%)
Admitting ward	
Department of Internal Medicine	17 (25.4%)
Surgical Intensive Care Unit	42 (62.7%)
Surgical ward	8 (11.9%)
Invasive procedure	
Total parenteral nutrition	27 (40.3%)
Invasive mechanical ventilation	21 (31.3%)
Hemodialysis	4 (6.0%)
Tracheostomy	3 (4.5%)
Central catheter	3 (4.5%)
Port catheter	1 (1.5%)
Vacuum-assisted closure	1 (1.5%)
Diagnoses and comorbidities	
Diabetes mellitus	25 (37.3%)
Hypertension	22 (32.8%)
Malignancy/hemopathy	16 (23.9%)
Kidney failure	13 (19.4%)
Cardiovascular disease	11 (16.4%)
Cerebrovascular accident	5 (7.5%)
Trauma	4 (6.0%)
Demyelinating disease	3 (4.5%)
Diagnosis of *Klebsiella pneumoniae* infection within the past 6 mo	15 (22.4%)
Prior exposure to ceftazidime–avibactam	2 (3.0%)
Prior carbapenem use	43 (64.2%)
Carbapenemase genes identified	
NDM	9 (13.4%)
OXA-48	7 (10.4%)
NDM + OXA-48	25 (37.3%)
Negative	26 (38.8%)

SD = standard deviation.

## 5. Distribution of clinical specimen types and antibiotic susceptibility

The 67 CAZ–AVI-resistant isolates were obtained from various clinical specimens including urine (31.3%), tracheal aspirates (29.9%), blood cultures (22.4%), wound (7.5%), bronchoalveolar lavage fluid (6.0%), and cerebrospinal fluid and peritoneal fluid (1.5% each).

The median colistin MIC among CAZ–AVI-resistant isolates was 2.0 μg/mL (IQR: 1.0–4.0). Colistin susceptibility was observed in 56.7% of these isolates. The median CAZ–AVI inhibition zone diameter was 10.0 mm (IQR: 9.0–11.0), confirming resistance according to EUCAST breakpoints (Table 2).

**Table 2:** Distribution of clinical specimens and antimicrobial susceptibility parameters (n = 67).

Variable	n (%) or median [IQR]
Specimen	
Urine	21 (31.3%)
Tracheal aspirate	20 (29.9%)
Blood	15 (22.4%)
Wound	5 (7.5%)
Bronchoalveolar lavage fluid	4 (6.0%)
Cerebrospinal fluid	1 (1.5%)
Peritoneal fluid	1 (1.5%)
Colistin MIC, μg/mL	2.0 [1.0–4.0]
Ceftazidime–avibactam inhibition zone, mm	10.0 [9.0–11.0]

IQR = interquartile range, MIC = minimum inhibitory concentration.

## 6. Discussion

Carbapenem-resistant *Enterobacterales* are classified among the World Health Organization’s “priority antibiotic-resistant pathogens,” published in 2017.^[15]^ Within the *Enterobacterales* order, *K pneumoniae* is the most frequently isolated species in healthcare settings and plays a pivotal role in the spread of carbapenemase genes. Owing to its ability to colonize hospital environments, *K pneumoniae* remains a leading cause of nosocomial infections.^[15]^ Accordingly, early identification of carbapenemase-producing microorganisms in both infected patients and asymptomatic carriers is essential to prevent outbreaks of healthcare-associated infections.^[16]^

In the present study, nearly one-third of the CRKp isolates originated from respiratory specimens. The majority of patients (62.7%) were hospitalized in the surgical intensive care unit, a setting where MDR gram-negative bacteria represent a serious threat due to their association with severe life-threatening infections. The patient population exhibited several common comorbidities, including diabetes mellitus (37.3%), hypertension (32.8%), and malignancy or hemopathy (23.9%). Invasive procedures such as total parenteral nutrition (40.3%) and mechanical ventilation (31.3%) were also frequent, reflecting a population at high-risk for MDR infections. Urine specimens accounted for the largest proportion of clinical specimens (31.3%), followed closely by respiratory specimens (29.9%). This distribution highlights the importance of monitoring resistance patterns in both urinary and respiratory tract infections caused by CRKp.

Routine reporting of resistance rates within healthcare institutions and the implementation of effective infection control measures are critical to guiding empirical therapy and informing antimicrobial policies. Given the lengthy development process for new antibacterial agents preserving the effectiveness of existing drugs as much as possible is imperative. In this context, CAZ–AVI has emerged as a promising option, demonstrating potent in vitro activity against *Enterobacterales* producing ESBL and AmpC β-lactamases across clinical isolates collected worldwide.^[17]^ In our cohort, CAZ–AVI resistance was detected in 21.5% of CRKp isolates, underscoring the growing challenge of managing CRKp infections, even with recently introduced therapeutic options.

The literature indicates considerable variability in CAZ–AVI susceptibility among CRKp isolates, reflecting temporal and geographic differences in carbapenemase gene distribution. For example, Zhang et al^[18]^ reported a resistance rate of 3.7% in China prior to the introduction of CAZ–AVI, whereas Krithika et al^[19]^ documented a resistance rate of 50.5% over a 6-month period in an Indian population. In a study from Bahrain investigating the risk factors associated with CAZ–AVI resistance in CRKp isolates, Ahmed et al^[20]^ reported a resistance rate of 22.4% and emphasized the need for enhanced infection control in patients with tracheostomies.

In Turkey, prior to the clinical availability of CAZ–AVI, resistance was identified in 9.8% of Enterobacterales isolates, suggesting its potential utility as an alternative to colistin for OXA-48 producers.^[21]^ National data provide further context: Özger et al^[22]^ reported a resistance rate of 4.7% among Enterobacterales isolates, supporting the efficacy of CAZ–AVI, particularly in pan-resistant strains. Similarly, Hoşbul et al^[14]^ observed a resistance rate of 7.3% among carbapenem- and colistin-resistant *K pneumoniae* isolates between 2018 and 2021, advocating for routine antimicrobial susceptibility testing informed by surveillance data. Our findings suggest an upward trend in CAZ–AVI resistance, likely attributable to expanded use. Alarmingly, 1 in 5 isolates was resistant to CAZ–AVI despite its restricted use.

The in vitro activity of CAZ–AVI against carbapenemase-producing Enterobacterales varies depending on the type of carbapenemase present. In our study, 61.1% of CAZ–AVI-resistant CRKp isolates harbored at least 1 carbapenemase gene. Specifically, blaOXA-48 was detected in 17.1% (n = 7), blaNDM in 21.9% (n = 9), and both genes coexisted in 61% (n = 25) of the isolates. These findings are consistent with the known spectrum of CAZ–AVI activity, which shows partial effectiveness against OXA-48 type carbapenemases but no activity against metallo-β-lactamases.

The high prevalence of NDM and OXA-48 in Turkey has been documented in various studies.^[23,24]^ These results align with global epidemiological trends showing that KPC-type carbapenemases are more common in the United States and Europe, while NDM predominates in South Asia, and OXA-48-like carbapenemases are endemic to the Mediterranean region, including Turkey. This global distribution is driven by factors such as international travel, medical tourism, and the overuse or misuse of antibiotics in both human and veterinary medicine, all of which have contributed to the worldwide spread of carbapenemase-producing Enterobacterales.^[4]^ Among the CAZ–AVI-resistant CRKp isolates in our study, the median colistin MIC was 2.0 μg/mL (IQR 1.0–4.0), with 56.7% (n = 38) classified as susceptible according to EUCAST breakpoints. Despite concerns regarding nephrotoxicity, colistin remains a viable therapeutic option for carbapenem-resistant enteric bacteria and demonstrates synergistic effects when combined with CAZ–AVI.^[25]^

CAZ–AVI is highly effective against OXA-48 producers.^[25]^ However, the increasing prevalence of metallo-β-lactamase producers presents a significant challenge to the efficacy of CAZ–AVI therapy, underscoring the need for further research into the synergistic potential of combining CAZ–AVI with metallo-β-lactamase inhibitors, such as aztreonam.^[3,26]^ Previous studies have identified prior carbapenem use as a risk factor for the development of resistance to CAZ–AVI. In our study, significant associations were observed between prior use of CAZ–AVI and diagnosis of a *K pneumoniae* infection within the past 6 months, as well as between prior carbapenem use and *K pneumoniae* diagnosis in patients with CAZ–AVI resistance. These findings highlight the role of antimicrobial pressure in the emergence of resistant *K pneumoniae* strains. Accordingly, in infections caused by ESBL-producing isolates, CAZ–AVI may be considered an alternative to carbapenem therapy.^[19]^ Carbapenemase-producing isolates have the potential to confer resistance to multiple drug classes, as they often harbor additional resistance elements alongside carbapenemase-encoding plasmids. Infection control measures and antimicrobial stewardship (AMS) remain the most critical strategies for mitigating carbapenem resistance. With the discovery of novel antibiotics, therapeutic approaches may vary according to the classification of carbapenemase-resistance mechanisms. A recent review emphasized that carbapenem resistance should be tested even in colonizing isolates if therapy with CAZ–AVI is considered.^[27]^ Further studies are warranted to support integration of molecular diagnostic methods into routine laboratory workflows for the management of carbapenem resistance.

Our study highlight a significant prevalence of NDM and OXA-48 carbapenemase genes among the isolates, with 37.3% of the samples harboring both genes, representing highly resistant phenotype contributing to multidrug resistance. Importantly, nearly 39% of the isolates did not carry these major resistance genes, suggesting that a substantial proportion of the bacterial population may still be susceptible to carbapenems, indicating a potential therapeutic window. These genetic markers provide crucial insights into the resistance landscape of *K pneumoniae* and are instrumental in guiding effective treatment strategies.

## 7. Limitations

While informative, this study has several limitations that may affect the generalizability of its findings. First, the sample size of 67 isolates, although sufficient for preliminary observations, may not capture the full spectrum of genetic diversity and resistance patterns present in broader populations. Additionally, the predominance of specimens from surgical intensive care units and specific specimen types introduces potential selection bias, limiting the applicability of these findings to other patient populations or healthcare settings.

In light of rising resistance rates, CAZ–AVI should be reserved for selected patients based on local epidemiology of carbapenemase genes and current treatment guidelines. Preventing carbapenem resistance through appropriate empirical antibiotic therapy guided by risk assessment, along with stringent infection control measures will support the continued viability of existing antibiotics as last-resort therapies.. Sustained surveillance, early detection through molecular diagnostics, and internationally coordinated containment efforts remain critical. Moreover, antimicrobial stewardship programs are vital for curbing inappropriate use of antibiotics and safeguarding the effectiveness of both current and emerging therapeutic agents.

## Author contributions

**Conceptualization:** Ipek Koçer.

**Data curation:** Ipek Koçer, Hadiye Demirbakan.

**Formal analysis:** Ipek Koçer, Hadiye Demirbakan.

**Funding acquisition:** Ipek Koçer.

**Investigation:** Ipek Koçer.

**Methodology:** Ipek Koçer.

**Writing – original draft:** Ipek Koçer.

**Writing – review & editing:** Hadiye Demirbakan.
